# The effect of complex training with traditional and variable resistance on lower limb muscle strength and swimming performance in competitive swimmers

**DOI:** 10.3389/fphys.2026.1884995

**Published:** 2026-07-07

**Authors:** Cuiqing Zhao, Liam Patrick Kilduff, Frank Nugent, Michael Keiner, Chao Chen, Zixiang Zhou

**Affiliations:** 1 School of Sports Science, Nantong University, Nantong, China; 2 Applied Sports, Technology, Exercise, and Medicine Research Centre, Faculty of Science and Engineering, Swansea University, Swansea, United Kingdom; 3 Department of Physical Education & Sport Sciences (PESS), University of Limerick, Limerick, Ireland; 4 German University of Health and Sport, Ismaning, Germany; 5 College of Physical Education, Dalian University, Dalian, China; 6 School of Humanities and Social Sciences, Shandong Medical and Pharmaceutical University, Yantai, China

**Keywords:** muscle strength, post-activation performance enhancement, swimming performance, variable-resistance complex training, vertical jump

## Abstract

**Purpose:**

To compare the effects of variable resistance complex training (VRCT) versus traditional complex training (TCT) on maximum strength, vertical jump ability and swimming performance in swimmers.

**Methods:**

Twenty-four male swimmers (age: 20.9 ± 1.1 years) were randomized to VRCT (n = 8), TCT (n = 8), or control (CON; n = 8) and completed a twice weekly training intervention over a 6-week period. Training programs involved alternating high-load resistance exercise with plyometric exercise within the same session. The VRCT group performed resistance exercises at 70% of 1-repetition maximum (1RM) + 0% to 23% of 1RM from band resistance with a 90-second rest interval, while the TCT group conducted resistance exercise at 93% of 1RM with a 4-minute rest interval. Maximum strength, vertical jump, start, turn and 100-m sprint performance were assessed pre- and post-intervention.

**Results:**

VRCT and TCT demonstrated significant increase in the 1RM back squat, countermovement jump and squat jump height (all *p* < 0.01). Furthermore, VRCT and TCT significantly improved turn (*p* < 0.01 and *p* < 0.05, respectively) and 100-m sprint time (both *p* < 0.01), whereas start time improved only in the VRCT (*p* < 0.01). Between groups, VRCT showed significant improvement in the start and turn times (*p* < 0.05) compared with CON.

**Conclusions:**

Both complex training modalities can be implemented to enhance lower limb strength, power and sprint performance. VRCT is more efficient to improve start and turn time in swimmers.

## Introduction

Swimming is a highly competitive sport in which even marginal time differences can change competition rankings. In competitive swimming, especially for short-distance events, performance is highly dependent on muscular strength and power, which has previously been shown to be a major determinant of swimming success (Crowley et al., 2017; Ruiz-Navarro et al., 2025). Previous studies have demonstrated that well-developed levels of lower-limb strength have a positive effect on performance improvement in swimmers (Gourgoulis et al., 2014; Keiner et al., 2021). The start and turn performance are two of the most important variables in competitive swimming (Veiga et al., 2016; Peterson Silveira et al., 2018; Born et al., 2024). Keiner et al (Keiner et al., 2021). indicated that 1RM squat performance, countermovement jump (CMJ) and squat jump (SJ) height were related to 15m start (*r* = -0.67 to -0.78) and turn (*r* = -0.54 to -0.75) performance. Based on these findings, it can be seen that there is a correlation between lower-body maximum strength, vertical jumping ability, and swimming performance. Therefore, effective resistance training may contribute to improved swimming performance.

Dry land resistance training is a common method to improve muscle strength in swimming, which can enhance swimming performance (Crowley et al., 2017). Traditional resistance training can improve lower limb strength and swimming performance. It is possible for swimmers to enhance swimming performance by increasing lower limb strength. For example, Lopes et al (Lopes et al., 2021). reported that eight weeks of dry land strength training, which included squat and CMJ exercises, enhanced CMJ (+6.8%, *p* = 0.013) and 50-m front crawl (+4.1%, *p* = 0.027) performance in national competitive swimmers. The maximum strength back squat training for 6 weeks significantly improved 25-m sprint time (Born et al., 2020). Nevertheless, Amaro et al (Amaro et al., 2017)reported that traditional strength training over a 6-week period was unable to induce a significant effect (*p* > 0.05) on 50-m swimming performance. Although some studies have confirmed the positive impact of traditional resistance training on muscle strength and swimming performance, it is not without limitations. Traditional resistance training is known as constant resistance training because the load lifted by an individual remains constant throughout the range of motion (Frost et al., 2010). Because the maximum load that can be lifted is generally dependent on a shortest range of motion called as the “sticking zone”, a constant load results in biomechanically disadvantageous positions to generate higher force and acceleration (van den Tillaar et al., 2014). As a result, traditional resistance training cannot assure that the muscle is sufficiently stimulated throughout the whole joint range of motion.

Traditional resistance and plyometric training are essential components of an athlete’s training program for the development of maximal force generation (MacDonald et al., 2012; Muniz-Pardos et al., 2022). However, both training methods have their own advantages and shortfalls for improving the lower limb strength and performance of swimming athletes, as previously described (Frost et al., 2010; van den Tillaar et al., 2014; Wang et al., 2022). Traditional complex training (TCT) may be an effective method since it allows for the execution of two training modes (resistance training and plyometrics) in a single session, alternating high-load resistance exercise with unloaded explosive exercise, thereby increasing maximal and fast strength output (Cormier et al., 2022). TCT is supported by post-activation performance enhancement (PAPE), that is defined as a short-term rise in force and power generation in muscle following a near-maximal voluntary contraction (Boullosa et al., 2020). Thus, high resistance can enhance maximal strength, while the PAPE effect may improve performance during subsequent plyometric exercises and also improve explosive power, consequently leading to greater training benefits. It has been reported that maximum strength and sprint performance can be improved to a larger extent by TCT compared to plyometric or heavy-resistance training alone (Hammami et al., 2019). However, there have been no investigations into the efficacy of TCT for competitive swimmers.

Variable resistance complex training (VRCT) adds additional resistance to the barbell by using elastic bands or chains to match the load and changing muscle strength potential. This causes the resistance exercise’s force-velocity curve to change, allowing for more velocity and power generated, attributed to the lower initial load (Heelas et al., 2021). It has been reported that VRCT may provide a higher training stimulus for improving strength and eliciting high levels of muscle activation than traditional resistance training (Heelas et al., 2021). It can be observed that variable resistance training not only overcomes the limitations of the “sticking zone” but also provides a relatively greater training load. Therefore, there would be reason to believe that VRCT can make up for the lack of traditional resistant and plyometric training. Recent studies revealed that both VRCT and TCT could effectively increase lower limb maximal strength, jumping performance and leg stiffness in athletes (Shi et al., 2022; Scott et al., 2023). Since VRCT may cause the muscles involved to exert near-maximum strength across the range of motion, it may be more suitable for swimming than other strength training methods. This is because start and turn performance require the limbs to maintain a greater force output throughout the entire range of joint motion. According to the formula: Drag (*F*) = Drag factor (*K*)× velocity squared(*v*^2^). Swimmers need to accelerate immediately by extending their lower limbs after the turn. During this low-to-high velocity acceleration phase, the resistance that must be overcome increases with velocity due to the change in velocity. Thus, the increasing resistance pattern in VRCT can match the increasing drag of accelerating movement in water. However, there is no experimental evidence suggesting two complex training methods are effective for swimming. Therefore, the purpose of this study was to investigate the effects of TCT in comparison to VRCT on physical and swimming performance in competitive swimmers. Compared to TCT, we hypothesized that VRCT would result in a greater gain in maximum muscle strength, jumping, and swimming performance.

## Methods

### Participants

Twenty-four national level elite male swimmers volunteered to participate in this study (mean ± SD; age 20.9 ± 1.1 years; height 181.5 ± 6.0 cm; body mass 79.8 ± 8.5 kg; training experience 8.9 ± 1.2 y). They have competed at the national level and hold National Level I athlete certification. The required sample size was calculated at prior using the software G*Power (3.1; University of Dusseldorf, Dusseldorf, Germany). Based on the study by Arazi et al (Arazi et al., 2020), the methods of repeated-measures ANOVA between factors (Number of groups: 3, Number of measurements: 2), with statistical power = 0.85, α level = 0.05, and effect size = 0.75. The total sample size of 18 subjects (6 per group) would be required. Participants were excluded if there were any muscular injury in the past six months. Swimmers exercised twice a day, six days a week for six months before the intervention, including at least 2 resistance training sessions. All the participants were informed about the intervention procedures, the potential risks and benefits of the experiment.

### Experimental design and procedures

**Figure 1:**
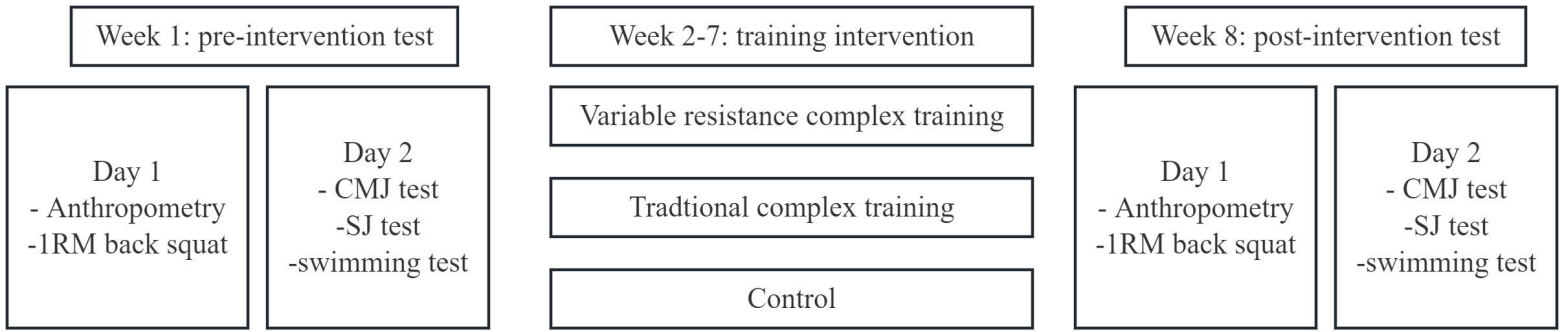
The experimental design and timeline of the study. CMJ, countermovement jump; SJ, squat jump; RM, repetition maximum.

This study implemented a randomized 6-week controlled trial design to investigate differences both within and between groups. The subjects were randomly divided into either TCT group (n = 8), VRCT group (n = 8), or a control (CON) group (n = 8). The VRCT and TCT groups participated in corresponding training interventions for six weeks, consisting of two sessions per week. The CON group did not engage in any resistance exercises. Maximal strength, CMJ, SJ, and swimming performance (start, turn, and 100-m sprint) were assessed before and after intervention (**Figure 1**).

Prior to the experiment, all participants were familiarized with the testing protocol and the resistance and plyometric exercises within the training program. After the familiarizing sessions, all participants performed performance assessments twice, with an interval of 48 hours between each assessment. Day 1 consisted of anthropometric characteristics (AM) and a maximum strength test (PM). Day 2 involved CMJ, SJ (AM), and swimming performances (PM). All measurements were conducted within a one-week period (Scott et al., 2023). The day following the maximal strength test, the specific contribution of variable resistance was determined using a force plate in the VRCT group, and participants then familiarize themselves with the VRCT program. All participants completed a general warm-up that comprised 5 minutes of jogging followed by 10–15 minutes of dynamic stretching and activation exercises before to each dry-land session. Data were collected from all participants after 6 weeks, with testing order, testing time, format, and testing duration consistent between the pre-training and post-training tests.

### Measures

#### Maximum strength test

According to established guidelines, the 1RM back squat was calculated (Baechle and Earle, 2008). After a 20-min warm-up that consisted of 5 min of jogging and 10 min of dynamic stretching, participants estimated their 1RM based on training experience, then completed three sets of warm-up exercises at 50%-80% 1RM. Following the warm-up, the participants completed 3–4 trials at their estimated 3RM, with 5 minutes of rest in between each trial. During the testing procedure, weight was progressively increased using small increments. The initial increase was set at 5 kg. If the lift was successful, subsequent increments were reduced to 2.5 kg until failure occurred. The final successfully lifted weight was then recorded as the 1RM. If the participant failed at the first 5 kg increase, the weight adjustment was shifted to a 2.5 kg increment for the subsequent attempt. During the back squat process, participants were required to bend their knees to approximately 90° and then return to an upright position until their legs were fully extended. The assessment has already showed excellent reliability [intraclass correlation coefficient (ICC) = 0.99] (Comfort and McMahon, 2015).

#### Vertical jump test

For the vertical jump testing, the prior procedures were used for the CMJ and SJ tests (Markovic et al., 2004). For each jump, three trials with a 2-minute recovery period were conducted, and the best performance was selected for further analysis. Prior to each jump test, participants performed two submaximal practice trials with a 1-minute rest interval. During the CMJ, participants were told to place their hands on their hips while making a downward movement to attain about 90° of knee flexion, with the objective of reaching the highest height possible in each jump. In the SJ test, participants were asked to remain in a static position with a 90° knee flexion angle for ~ 2 s before jumping, with no preceding movement. During each jump, participants were encouraged to make every attempt to jump as high as possible. The jump test was performed on a contact mat (Smart Jump; Fusion Sport, Coopers Plains, Australia). The intraclass correlation coefficient (ICC) was 0.94 for the CMJ, 0.96 for the SJ.

#### Swimming performance

Swimming tests were performed in a 50-meter indoor pool with water temperatures of 27.5 °C and a relative humidity of 67%. All participants completed a standard warm-up program consisting of a 600-m front crawl and three 50-m front crawl trials at incrementally higher speeds. Each swimmer completed two 100-m maximal sprints with a 20-minute recovery period, and the best value was used for further analysis. During the 20-minute recovery period, participants wore clothes to rest and maintain their body temperature, and they performed stretching and warm-up exercises before the second test. According to prior methods (Born et al., 2020), a standardized starting procedure was used. Swimmers took their places on the starting block (OSB 11; Omega, Swiss Timing LTD, Switzerland) and were given instructions to “take your mark” before the starting device sounded. The start signal is heard to the swimmers and is visible to the cameras as a flashing light. The block was placed in the third lane, 6.6-m from the side wall to which the camera system was set up. Swimming performance was recorded through a camera system (cameras: Sony FDR-AX45; Sony, Tokyo, Japan) with 3 cameras (1.5 m above the water surface at 15, 35, and 45 m). The start and turn times were measured by the performance of the 100-meter sprint test. The start performance was measured when the head of the swimmers passed the 15-m mark, and the turn performance was assessed by the time lag between reaching the 45-m mark and the 15-m mark of the following split (Morais et al., 2019). The start and turn performance were determined using video analysis system software (Kinovea, version 0.8.15, Joan Charmant and Contrib., kinovea.org).

### Determination of individual training loads

Following previously known procedures (Scott et al., 2018), the variable resistance from the latex bands was calculated for VRCT. Briefly, the participant stood on a force plate (Kistler, model 9290AA, Winterthur, Switzerland). The bar was placed on their back, and both their mass and that of the bar were recorded. The bands (Decathlon) were anchored to the bar, and the participants stood at the end of the range for each squat, and their mass was also recorded. The difference between these two measurements was used to determine band tension. This procedure was repeated with bands of varying tension until the variable resistance at the end range of each exercise reached 23% 1RM. Finally, the total resistance at the end position of the exercise reached 93% 1RM.

### Training program

Complex training was conducted twice a week for six weeks, with a rest period of 48 to 96 hours in between each session. Complex training consisted of a set of back squats followed by a set of plyometric jumps (**Table 1**). We chose these exercises from **Table 1** because they were particularly beneficial for swimmers (Crowley et al., 2018) and target key lower-body movement patterns that are transferrable to swimming performance (Sammoud et al., 2019). Between training groups, the volume load of the exercises provided (sets × repetitions × barbell weight) was consistent; however, the barbell load and rest interval varied. Resistance exercises for TCT were completed at 93% of 1RM, separated with a 4-minute recovery period according to previously published methods (Scott et al., 2023). Since this load commonly corresponds to an athlete’s 3RM, which aims for maximum strength, a 2- to 5-minute rest period was recommended to experience the greatest strength training gains. For VRCT, the resistance exercises were carried out at 70% of 1RM + 0% (bands relaxed state) to 23% (bands stretched state) of 1RM from band resistance with a 90-second rest interval because this was considered the shortest time for eliciting the PAPE effect (Scott et al., 2018). The participants were instructed to perform all exercises at maximal intended velocity. Researchers and coaches provided complete supervision for each training session. During intervention period, the participants swam about 355 km, the equivalent to a mean value of 59.17 km per week and 4.93 km per training unit. All participants performed the same swimming training without any additional strength training. The swimming training included low aerobic (~ 77%), technical skills (~21%), and speed swimming sets (~2%).

**Table 1:** Complex training programs for VRCT and TCT.

	VRCT			TCT		
Complex pairs	Sets × reps	Load	Interval	Sets × reps	Load	Interval
1a. Back squat	3 × 3	70% + 0% – 23% 1RM	90-s	3 × 3	93% 1RM	4-min
1b. CMJ	3 × 6	Body weight		3 × 6	Body weight	
2a. Back squat	3 × 3	70% + 0% – 23% 1RM	90-s	3 × 3	93% 1RM	4-min
2b. DJ (40 cm)	3 × 6	Body weight		3 × 6	Body weight	
3a. Back squat	3 × 3	70% + 0% – 23% 1RM	90-s	3 × 3	93% 1RM	4-min
3b. Long jump	3 × 6	Body weight		3 × 6	Body weight	

VRCT, variable-resistance complex training; TCT, traditional complex training; CMJ, countermovement jump; DJ, drop jump; RM: repetition maximum.

### Statistical analysis

All data are presented as mean ± standard deviation. The Shapiro-Wilk test for data normality was applied. A 3 (groups: VRCT, TCT and CON) × 2 (time: pre-intervention and post-intervention) repeated measures ANOVA was used to examine group × time interactions and within-group changes. Where significant main effects and interactions were identified, Bonferroni adjustments were applied as appropriate. Effect sizes were calculated by partial eta squared (η*_p_*²) values for two-way ANOVA with repeated measures, and values interpreted as: minimum if 0.04 < η*_p_*² ≤ 0.25; moderate if 0.25 < η*_p_*² ≤ 0.64; and strong if η*_p_*² > 0.64. Based on previous study (Fone and van den Tillaar, 2022), percent change and between-group effect size were calculated to compare the effects of different interventions. One-way ANOVA was used to compare the percentage changes. The effect size of the difference between the groups was calculated by Cohen’s d (*d*) (trivial: *d* < 0.2, small: 0.2 ≤ *d* < 0.5, moderate: 0.5 ≤ *d* < 0.8, large *d* ≥ 0.8). Statistical significance level was set at *p* < 0.05. All data analyses were analyzed using SPSS 26.0 software (IBM Corp. Amork, NY) and PRISM (GraphPad Software, Inc. Version 9.0.2 for Windows).

## Results

**Figure 2:**
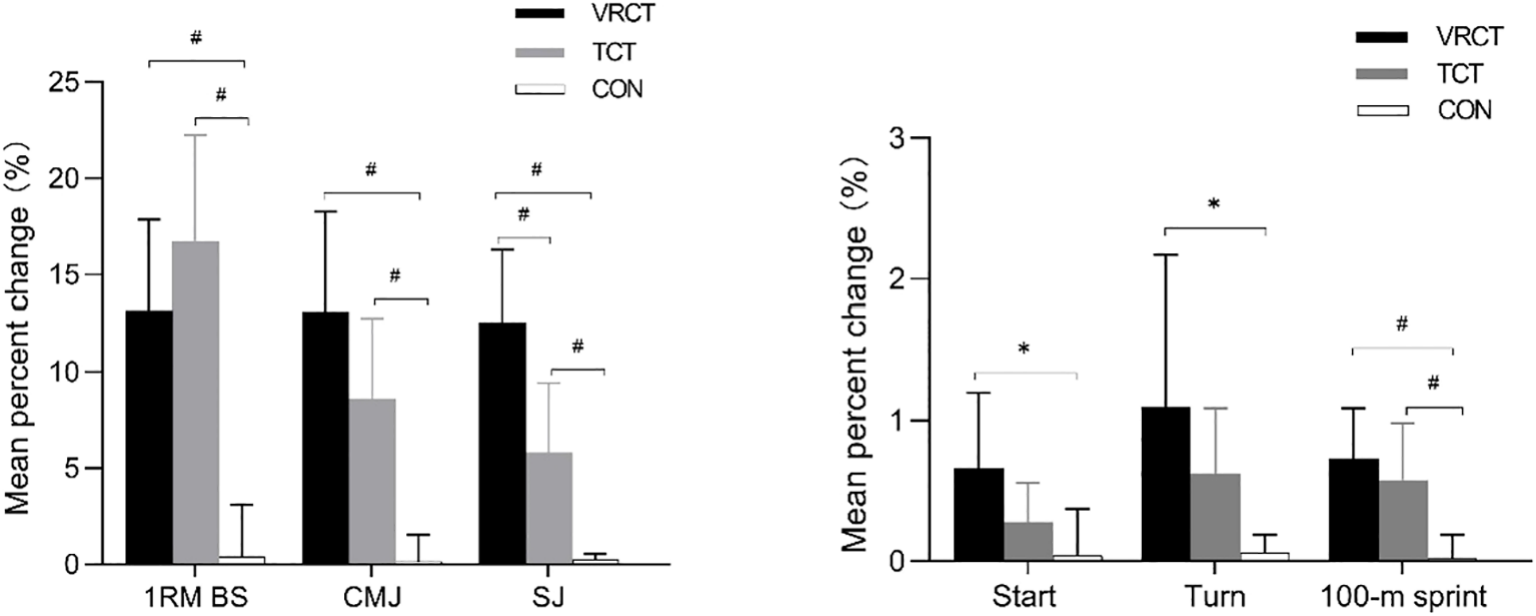
Mean percent change between groups differences in strength, vertical jump and swimming performance. RM, repetition maximum; BS, back squat; VRCT, variable-resistance complex training; TCT, traditional complex training; CON, control; RM, repetition maximum; CMJ, countermovement jump; SJ, squat jump. ^*^Significant differences in percent change between groups (*p* < 0.05). ^#^Significant differences in percent change between groups (*p* < 0.01).

No significant (*p* > 0.05) differences were found in the dependent variables at baseline between the groups. The mean values, within-group changes, and effect sizes in the performance assessment are displayed in **Table 2**. **Figure 2** presents the between-group differences in percent change. The repeated measures ANOVA revealed a significant group × time interaction (F = 26.308, *p* < 0.001, η*_p_*² = 0.715) for the back squat 1RM. VRCT and TCT groups showed a significant (*p* < 0.01) within-group improvement following intervention. No significant difference was observed in the CON group. Both VRCT (*p* < 0.01) and TCT significantly (*p* < 0.01) improved 1RM back squat compared with CON.

**Table 2:** Comparison of strength, vertical jump and swimming performance, within-group change scores and their corresponding 95% CIs at preintervention and postintervention.

	VRCT	TCT	CON
1RM back squat (kg)			
Pre	75.79 ± 13.96	81.92 ± 9.78	80.11 ± 13.05
Post	87.58 ± 17.22^#^	95.56 ± 11.23^#^	80.23 ± 11.87
Change score (95% CI)	11.79 (8.08, 15.51)	13.64 (9.93, 17.36)	0.12 (-3.59, 3.85)
Cohen’s *d*	0.75	1.30	0.01
CMJ (cm)			
Pre	37.81 ± 6.08	38.15 ± 4.93	37.97 ± 4.55
Post	42.73 ± 6.77^#^	41.32 ± 4.37^#^	38.04 ± 4.82
Change score (95% CI)	4.92 (3.65, 6.18)	3.17 (1.90, 4.43)	0.08 (-1.19, 1.34)
Cohen’s *d*	0.76	0.68	0.01
SJ (cm)			
Pre	33.02 ± 3.62	34.01 ± 4.76	34.64 ± 4.66
Post	37.09 ± 2.83^#^	35.90 ± 4.93^#^	34.65 ± 4.70
Change score (95% CI)	4.08 (3.31, 4.85)	1.89 (1.12, 2.66)	0.02 (-0.75, 0.79)
Cohen’s *d*	1.25	0.39	0.002
Start performance (s)			
Pre	7.24 ± 0.15	7.22 ± 0.12	7.18 ± 0.13
Post	7.19 ± 0.17^#^	7.20 ± 0.13	7.18 ± 0.14
Change score (95% CI)	-0.05 (-0.07, -0.02)	-0.02 (-0.04, 0.001)	-0.002 (-0.03, 0.02)
Cohen’s *d*	0.31	0.16	0.001
Turn performance (s)			
Pre	10.89 ± 0.62	10.81 ± 0.55	10.71 ± 0.56
Post	10.77 ± 0.55^#^	10.74 ± 0.54*	10.70 ± 0.57
Change score (95% CI)	-0.12 (-0.18, -0.07)	-0.07 (-0.13, -0.01)	-0.01 (-0.07, 0.05)
Cohen’s *d*	0.20	0.13	0.02
100-m sprint (s)			
Pre	54.00 ± 1.16	53.89 ± 0.98	53.75 ± 1.02
Post	53.60 ± 1.19^#^	53.58 ± 0.98^#^	53.74 ± 0.99
Change score (95% CI)	-0.39 (-0.52, -0.27)	-0.31 (-0.44, -0.19)	-0.01 (-0.14, 0.12)
Cohen’s *d*	0.34	0.32	0.01

VRCT, variable-resistance complex training; TCT, traditional complex training; CON, control; RM, repetition maximum; CMJ, countermovement jump; SJ, squat jump. Data are presented as mean (SD) and 95% CI.

^*^ Significantly change from pre-training to post-training (*p* < 0.05). ^#^Significantly change from pre-training to post-training (*p* < 0.01).

The statistical analysis showed there was a significant group × time interaction (F = 25.430, *p* < 0.001, η*_p_*² = 0.708) for the CMJ test. VRCT and TCT showed a significant within-group improvement following training interventions. However, no change was found in the CON group. Both VRCT (*p* < 0.01) and TCT (*p* < 0.01) significantly improved CMJ performance compared with CON.

Statistical analysis revealed a significant group × time interaction (F = 56.162, *p* < 0.001, η*_p_*² = 0.842) for the SJ test. VRCT and TCT groups showed a significant (*p* < 0.01) with-group improvement after training. In comparison to CON, the percent change was significantly greater following VRCT (*p* < 0.01) and TCT (*p* < 0.01). Furthermore, the SJ improved significantly (*p* < 0.01) more following VRCT compared to TCT.

For the start performance, there was a significant group × time interaction (F = 4.997, *p* = 0.017, η*_p_*² = 0.322). VRCT exhibited a significant (*p* < 0.01) within-group change. No significant difference was observed for the TCT and CON. Moreover, VRCT (*p* < 0.05) significantly improved start performance compared with CON.

The repeated measures statistical analysis indicated a significant group × time interaction (F = 4.378, *p* = 0.026, η*_p_*² = 0.294) for turn performance. In the VRCT (*p* < 0.01) and TCT (*p* < 0.05), turn performance was significantly changed. However, no change (*p* > 0.05) was found in the CON group. VRCT (*p* < 0.05) significantly improved turn performance compared with CON.

A significant group × time interaction effect (F = 11.050, *p* = 0.001, η*_p_*² = 0.513) was observed for the 100-m sprint performance. VRCT and TCT groups indicated a significant (*p* < 0.01) within-group enhancement following intervention. No significant difference was observed in the CON. Moreover, compared to the CON group, the sprint performance was significantly better following VRCT (*p* < 0.01) and TCT (*p* < 0.01).

## Discussion

The purpose of this study was to compare the effects of 6-weeks VRCT and TCT on neuromuscular adaptations and swimming performance in competitive swimmers. To our knowledge, this is the first study to investigate the effects of complex training on swimming performance. The main finding of the present study was that VRCT and TCT resulted in similar adaptations to maximum strength, CMJ and SJ height. This study demonstrates that both VRCT and TCT lead to a significant improvement in the turn time and 100-m swimming time but an improvement in start time was only observed in the VRCT group. Furthermore, after the intervention, the VRCT group exhibited a greater percentage change in start and turn performance compared to the CON.

The VRCT and TCT groups both experienced significant increases in maximum strength (VRCT + 11.79kg, TCT + 13.64kg), but there were no between-group differences. The present findings are similar to previous studies and indicates that VRCT and TCT result in comparable maximal strength improvements after 6-week intervention, and TCT exhibited a greater mean precent change (MacDonald et al., 2012; Scott et al., 2023). Prior research has demonstrated that the cross-sectional area of type II muscle fibers in the vastus lateralis is significantly greater following 6-week complex training compared to before the intervention (Stasinaki et al., 2015). Thus, the enhancement of maximal strength can be attributed to neural adaptations and myofiber contractile properties. This is advantageous for swimmers, as improvements in lower-limb strength have a positive effect on performance in swimmers (Gourgoulis et al., 2014; Keiner et al., 2021). Lower limb strength is needed to enhance leg kick performance, thus keeping the body in a streamlined position and increasing swimming velocity (Gourgoulis et al., 2014).

Both VRCT and TCT significantly improved CMJ and SJ performance. However, as seen from the effect size and change score, the increase in SJ may be more evident in the VRCT group. In comparison to the TCT and CON groups, this study revealed a significant increase for the SJ in the VRCT group. Our findings are consistent with prior study, which shown that variable resistance exercises are preferable to traditional complex training for increasing vertical jump ability (Loturco et al., 2022). One reasonable explanation is that the greater changes in vertical jump tests observed in the VRCT could be associated with the higher neuromuscular adaptations caused by the increased and progressive rate of elastic tension produced throughout the entire range of motion, which requires players to accelerate continuously during the concentric phase (Loturco et al., 2022), therefore allowing for greater transference to specific performance (Cormie et al., 2011). On the other hand, greater muscle fiber adaptations may result from a faster velocity in the biomechanically disadvantageous position (sticking point) caused by the decreased loading during the initial concentric stage of VRCT (Kompf and Arandjelović, 2016). In vertical jump tests, higher acceleration will result in higher takeoff velocity, leading to higher height (Loturco et al., 2015). As a result, the cumulative effect of continuously accelerative exercises may explain the advantage of VRCT on the CMJ and SJ. The improvement in vertical jump performance is beneficial for competitive swimmers. It has been reported that there is a strong correlation between CMJ, SJ height and start/turn performance (*r* = -0.65 to -0.78) (Keiner et al., 2021). Some studies have also found a correlation between CMJ height and starting performance (*r* = - 0.62 to -0.69) (West et al., 2011; García-Ramos et al., 2016). In summary, an increase in leg pushing force at the starting block level (start) and at the wall level (turn) owing to an increase in lower limb maximal strength might explain the current findings.

There is evidence to suggest that start and turn performance showed significant within-group improvement in both VRCT and TCT. However, only VRCT exhibited a significantly greater mean percent change in start and turn performance compared to CON. The improvement in start and turn time might be related to the enhancement of SJ and CMJ, as many studies have found that vertical jump ability and start/turn performance are significantly correlated (García-Ramos et al., 2016; Keiner et al., 2021). Bishop et al. reported that 8 weeks of plyometric training (squat jump, depth jump, etc.) can significantly enhance the 5.5-m starting performance (3.88s to 3.29s) in adolescents (Bishop et al., 2009). Maximum strength training can also improve starting performance. Born et al. found that 6 weeks of maximal strength training (heavy-loaded back squat and deadlift exercise) can reduce 15-m start time (7.28s to 7.21s) significantly in junior swimmers (Born et al., 2020). There were no previous studies investigating the impact of increasing lower limb strength on turning performance. However, Keiner et al. demonstrated a high correlation between CMJ,SJ and turn performance (*r* = -0.75 to -0.65, respectively) (Keiner et al., 2021). These studies suggest a significant contribution of lower limb muscle strength to the start and turn performance of swimmers. The start and the turn represent the moments during the race when the highest acceleration is achieved due to the swimmer having contact with a fixed structure (i.e., the wall/starting blocks) (Keiner et al., 2021). We hypothesize that in the initial phase of the turn, the extensor muscles of the lower limbs are working eccentrically. In order to produce greater acceleration, the eccentric phase is followed by a concentric phase in which the hip, knee, and ankle are extended. The resistance mode given by the elastic bands could have increased elastic energy storage during the eccentric phase, which may have contributed to the concentric phase by releasing more kinetic energy (Hostler et al., 2001; Wyland et al., 2015). We believe this may be due to the greater velocity induced by VRCT during the eccentric phase, which could elicit a muscle “stretch reflex” effect. However, we unfortunately did not measure the barbell movement velocity. Furthermore, the similarities in both the progressive resistance characteristic and the movement pattern between VCRT and in-water turns may explain these results. Because a swimmer is subjected to the resistive forces of water, that is, drag depending on a drag factor and the swimming velocity squared. Drag (resistance) = Drag factor × velocity squared. Thus, swimmers need a powerful push-off after a turn to overcome the increased drag resulting from higher velocity. Similarly, VRCT also needs to overcome increased resistance.

Both VRCT and TCT also significantly improved 100-m swimming times. In this study, the improvement in the 100-m swimming performance might be attributed to an increase in lower body strength, thereby enhancing the start and turn performance. Although greater leg strength and jump ability could lead to improved leg kick performance (Gourgoulis et al., 2014), the mean swimming velocity generated during leg kick velocity was not directly measured between 15 and 45m and is, thus, speculative. A previous study noted that 6 weeks of low-limb plyometric training significantly enhanced 50-m (1.25m·s^-1^ to 1.29m·s^-1^) and 400-m (0.92m·s^-1^ to 0.96m·s^-1^) front crawl speed (Potdevin et al., 2011). Garrido et al. indicated that strength training such as leg extension and CMJ can significantly (*p* < 0.01) improve the percent change in the 25-m and 50-m swimming speeds (Garrido et al., 2010). In conclusion, both VRCT and TCT have positive effects on 100-m sprint swimming. Although VRCT and TCT only improved their 100-m sprint performance by -0.39s and -0.31s, respectively, this is still noteworthy for short-distance events since winning margins in sprint swimming can be as small as 0.01s. However, it is worth noting that the execution of a shorter rest interval in VRCT is highly practical during the actual training process.

Despite these new evidences provided by the current experiment, this study has some limitations. The production of PAPE is greatly individualized, and the training factors (rest interval) implemented may not have generated an optimal response in all swimmers (Crewther et al., 2011). The sample size of this study was small; nonetheless, recruiting active athletes for training intervention research is challenging. The lack of appropriate resistance training in the CON may have amplified the intervention effects observed in this study. Future research should investigate the impact of complex training with different modalities and loads on competitive swimming in larger sample sizes. Additionally, future research needs to focus on the effects of performing VRCT to the upper limbs.

### Practical applications

This study indicates that coaching staff can incorporate VRCT into the resistance training programs of elite swimmers. This is because VRCT can induce favorable adaptations for lower limb explosive power, which is a key indicator for improving start and turn performance, thereby enhancing swimming sprint performance. Additionally, TCT shows greater benefits in improving lower limb maximum strength. According to the force-velocity curve, increases in maximum muscle strength contribute to further improvements in explosive power performance. Therefore, coaches can alternate between the two types of resistance training in their practice.

## Conclusion

The outcomes of this study suggest that both complex training modalities can improve lower limb strength, vertical jump and 100-m sprint performance in competitive swimmers. These significant enhancements occurred at different magnitudes following 6 weeks of training. Additionally, we found that VRCT may provide additional benefits to start and turn performance. Thus, strength and conditioning coaches should execute complex training protocols according to the training targets.

## Data Availability

The raw data supporting the conclusions of this article will be made available by the authors, without undue reservation.
